# Relationship between socioeconomic inequality and multimorbidity progression in UK Biobank data

**DOI:** 10.1038/s43856-026-01607-5

**Published:** 2026-05-05

**Authors:** Jie Zhang, Lasse Bjerg, Susanne Boel Graversen, Henrik Støvring, Christina C. Dahm, Luke Johnston, Bendix Carstensen, Daniel R. Witte

**Affiliations:** 1 Steno Diabetes Center Aarhus, Aarhus, Denmark; 2 Department of Public Health, Aarhus University, Aarhus, Denmark; 3 Department of Biomedicine, Aarhus University, Aarhus, Denmark; 4 Steno Diabetes Center Copenhagen, Copenhagen, Denmark

**Keywords:** Epidemiology, Cardiovascular diseases

## Abstract

**Background:**

Socio-economic status (SES) is associated with many adverse health outcomes, yet it remains unclear how SES relates to the rate at which people accumulate long-term conditions (LTCs) over time. We investigated this relationship between SES and disease accumulation using longitudinal disease tracking data.

**Methods:**

We analyzed data from the UK Biobank study (n = 502,368, median age 58 years [range 37–73], 46% male at baseline) with a median follow-up of 15.8 years. We tracked accumulation of 80 specified LTCs (identified from hospital records using ICD-10 codes). Multistate models were used to estimate the transition rates between SES and incremental morbidity states (i.e., 0 to 1 LTC, 1 to 2 LTCs, until 7 to 8 + LTCs), with death as the absorbing state. SES indicators included education level, family income, Townsend Deprivation Index, and Index of Multiple Deprivation. The models were adjusted for age, sex, ethnicity, calendar year, current number of LTCs, and lifestyle factors.

**Results:**

Over 7.5 million person-years of follow-up, we observe a clear socioeconomic gradient in disease accumulation rates. All four SES indicators are associated with accelerated morbidity progression and mortality. The socioeconomic gradient is evident across all transition stages but notably stronger for the initial transition from health to the first LTC, where the lowest income group has a 71% higher transition rate (95% CI: 1.67–1.76).

**Conclusion:**

Disadvantaged SES is associated with higher rates of progression to subsequent morbidities. These findings show the lasting impact of socioeconomic disadvantages on the widening health gap in later adulthood.

## Plain language summary

Socio-economic status (SES) is associated with many adverse health outcomes in adulthood. However, these associations have generally been studied for a single disease, and it remains unclear how SES is associated with the rate at which people develop and accumulate long-term conditions (LTCs) over time. We studied over 500,000 UK adults for nearly 16 years, tracking disease accumulation from 80 LTCs. We found that people with lower SES developed LTCs more rapidly than those with higher SES. This pattern was consistent for all LTC states, with stronger associations observed with early disease. Even after accounting for lifestyle factors like smoking and exercise, lower SES remained strongly linked to faster disease accumulation. These findings show the lasting impact of socioeconomic disadvantages on the widening health gap in later adulthood, suggesting targeted interventions for those with lower SES may be beneficial.

## Abstract (web summary)

Zhang et al. study how socioeconomic status is associated with the rate at which people develop multiple long-term conditions using multistate models. Over 7.5 million person-years of follow-up, a clear socioeconomic gradient in disease accumulation rates, particularly at early stages of disease progression is seen.

## Introduction

Multimorbidity, referring to the co-occurrence of at least two long-term chronic conditions (LTCs) in the same individual^1^, is an increasing challenge in aging societies^2^. The prevalence of multimorbidity has risen and affects around 37% of adults globally^3^, although this estimate depends highly on the definition. Aging is the strongest risk factor for multimorbidity, reflecting chronic dysregulation of multiple organ systems^2^. A faster accumulation of multimorbidity may in turn serve as a clinical indicator of progressive loss of resilience and homeostatic multisystem dysregulation, which are key features of the biological aging process^4^. Furthermore, individuals with multimorbidity typically experience declining physical function and quality of life^5^, increased hospitalizations^6^, treatment burden, and mortality^7^.

Socioeconomic status (SES) is an established risk factor for multiple chronic diseases^8^, yet most existing evidence is derived from cross-sectional studies^9^. It remains unclear whether SES primarily influences the initial occurrence of chronic conditions or continues to shape their subsequent accumulation over time. Addressing this question requires moving beyond binary definitions, such as the presence of two or more LTCs at a given time point, which provides a coarse measure of a highly complex process. Emerging research indicates that simple diseases count inadequately capture the true burden of multimorbidity^9^. Instead, the rate at which conditions accumulate appears to be a stronger predictor of adverse outcomes such as disability and mortality^10^.

Longitudinal data are essential for capturing the dynamic nature of multimorbidity trajectories, including how rapidly individuals accumulate conditions over time. Recent population-based studies have begun to identify distinct longitudinal trajectories of multimorbidity as functions of different chronic disease accumulation rates^11,12^. Advanced statistical approaches, such as multistate Markov models can quantify the transition rates between different disease states while accounting for concurrent conditions, and evaluate how various factors influence these transitions^13,14^. These analyses can help identify modifiable risk factors that could prevent or slow multimorbidity progression and reveal shared biological mechanisms, that potentiate the development of further LTCs when prior LTCs are present.

In this work, we hypothesize that adverse SES accelerates both the onset of the first chronic condition and the subsequent accumulation of additional conditions. Because SES encompasses multiple dimensions, including education, income, and area-level deprivation, different SES indicators may have distinct effects on disease accumulation, potentially reflecting different pathways through which social advantage or disadvantage operates. We further hypothesize that different SES indicators may have varying effects depending on a person’s current disease stage. Multistate modeling was applied to quantify how various SES factors influence the rate of disease accumulation and mortality risk, while accounting for existing LTCs. We extended previous research by tracking disease accumulation up to more than 8 LTCs, providing a more comprehensive understanding of how disease accumulation across different socioeconomic groups.

Over 7.5 million person-years of follow-up, lower SES is associated with faster accumulation of LTCs and higher mortality risk across disease stages. All four SES indicators are associated with increased rates of disease accumulation, although the strength of association varies by LTC states.

## Methods

### Study design, setting, and participants

UK Biobank (UKB) is a population-based health research resource data consisting of ~500,000 people, aged between 37 years and 73 years, who were recruited between 2004 and 2010 from across the UK. Participants provided a range of information on socio-demographic characteristics, and lifestyle factors via questionnaires and interviews; Anthropometric measures, blood pressure readings, and samples of blood were also taken. The study website provides information about available data (http://www.ukbiobank.ac.uk). A full detailed description of the study design, participants, and quality control methods has been published previously^15–17^. We followed the Strengthening the Reporting of Observational Studies in Epidemiology (STROBE) guidelines^18^.

### Outcome-multimorbidity

There is no standard approach to operationalize the measurement of multimorbidity^19^, we therefore selected 80 specific LTCs based on Delphi recommendations^20^, the study by Barnett^21^, and other established comorbidity indices^22,23^. The occurrence of a health condition was defined by hospital episode statistics (HES) records, which uses the International Classification of Diseases 10th revision (ICD-10) diagnosis code and Office of Population Censuses and Surveys (OPCS-4) for procedures.

LTC categories included blood and blood-forming organs, circulatory system, digestive system, endocrine, nutritional and metabolic system, genitourinary system, infectious and parasitic diseases, mental health, musculoskeletal system, neoplasms, nervous system, respiratory system and symptoms. Detailed definitions of the 80 LTCs are provided in Supplementary Data 1.

We identified the first recorded diagnosis of each condition and used these data to construct participants’ chronological sequence of disease accumulation. Morbidity was defined as the presence of any LTC included in our study list. During follow-up, we tracked the cumulative number of LTC diagnoses for each participant. Rather than applying a binary cutoff to classify multimorbidity, we modeled morbidity as a dynamic process, allowing individuals to transition sequentially between morbidity states (e.g., 0 → 1 LTC, 1 → 2 LTC, …, 7 → 8+ LTCs) throughout follow-up.

All participants were followed from enrollment until death, loss to follow-up, or the end of the study (December 31, 2023), whichever came first.

### Demographic characteristics and lifestyle factors

Information on SES and lifestyle factors was collected through questionnaires at baseline. We examined both individual-level (education, family income) and area-level of indicators of SES (Townsend deprivation Index [TDI] and Index of multiple deprivation [IMD]). Education was categorized as college or above; A levels; O levels, GCSEs or equivalent; NVQ, HND, HNC, or equivalent; other professional qualifications; or none of the above (equivalent to less than high school diploma and CSEs or equivalent). Annual family income was reported in five groups: <£18,000, £18,000–£30,999, £31,000–£51,999, £52,000–£100,000, and >£100,000. TDI is a measurement of material deprivation, derived from census indicators of unemployment, non-car ownership, non-home ownership, and household overcrowding^24^. In the UKB, TDI scores are provided at the postcode level and categorized into deciles ranging from 1 (least deprived) to 10 (most deprived). IMD is a composition of income, employment, education, crime, barriers to housing and services, and neighborhood living environment^25^. For analysis, we converted TDI and IMD to quintiles for our analyses, with higher values indicating greater deprivation.

Lifestyle factors were assessed at baseline and scored based on adherence to recommended health guidelines according to previous studies^26,27^ and World Health Organization (WHO). For each factor, 1 point indicated an unhealthy behavior and 0 point indicated a healthy behavior. Smoking status was categorized as unhealthy for current or former smokers, and healthy for non-smokers. Alcohol consumption was considered unhealthy for those consuming alcohol one or more times per month to daily, and healthy for those who never drank or drank only on special occasions. Physical activity was measured using metabolic equivalent task (MET) minutes per week from a self-reported questionnaire, with unhealthy levels defined as below the median MET, and healthy levels equal to or above the median. Sleep duration was classified as unhealthy for less than seven hours per night and healthy for seven or more hours. Dietary health was assessed based on adherence to recommendations for ten food components according to previous UK Biobank studies, with unhealthy diet defined as meeting fewer than five recommendations, and healthy diet as meeting recommendations for five or more components. A composite lifestyle score was calculated by summing these five factors, ranging from 0 (healthiest) to 5 (least healthy). Height was measured (seca 202 stadiometer; seca) and weight was measured to the nearest 0.1 kg (BC-418 MA body composition analyzer; Tanita Corp). Body mass index (BMI) was derived from weight in kilograms divided by height in meters squared. BMI was then categorized according to WHO recommendations as: normal weight (<24.9 kg/m^2^), overweight (25.0–29.9 kg/m^2^), or obesity (≥30 kg/m^2^). Other demographic characteristics include age at baseline, sex (male/female), and ethnicity (White/Black/Asian/other).

### Statistical analysis

All sociodemographic characteristics and risk factors at baseline were summarized using means and standard deviations (SD), median and interquartile range (IQR), or counts and percentages as appropriate.

### MLTC accumulation rate

We employed a multistate modeling approach to analyze the cumulative number of LTCs. At cohort entry, participants were classified according to their baseline number of LTCs (ever recorded in available HES data). Throughout the follow-up period, we tracked each participant’s accumulation of new LTCs. A 12-month bandwidth was allowed for the diagnosis after entering the cohort (if an ICD diagnosis occurred within the first year of follow-up, it was still considered present at baseline).

For each participant, we created detailed longitudinal records including birth date, cohort entry and exit dates, dates of each new LTC diagnosis, and death date if applicable. The follow-up time was split at each transition point (new LTC diagnosis). Within these diagnosis-based intervals, we further subdivided the data by single calendar year, allowing us to model the rate of progression between states while accounting for temporal variations. Death was treated as an absorbing state, accessible from any other state. Multistate models were fitted to estimate transition rates between successive LTC states using the time-split data. Consistent with previous work, we assumed that LTCs remained present after the first occurrence. For several LTCs occurring on the same day for a given participant (Supplementary Data 2), we imposed an arbitrary ordering by adding 1-day increments within each same-day cluster, so that each diagnosis had a unique date for multistate model specification. This tie-breaking procedure does not affect the total number of events but avoids simultaneous transitions in the model.

The analysis used a Lexis object framework^28,29^ with three time dimensions: calendar time, current age, and time since enrollment. The sequential progression of LTCs were represented using a transition matrix (Supplementary Data 3). Because the number of transitions with eight or more LTCs was small, we grouped these states into a single category 8+LTCs, to avoid ill-determined transition rates. Using maximum likelihood estimation, we modeled the sequential progression of LTCs through eight distinct transitions (0 → 1 LTC, 1 → 2 LTC, up to 7 → 8+ LTCs).

We also modeled mortality risk from each morbidity state, examining transitions from each level of LTCs to death. The transition rates between LTC states were modeled using Poisson regression. Our results are therefore state transition rates and transition rate ratios. We denote these as Transition Rate Ratios (TRR) for transitions between LTC states, and Mortality Rate Ratios (MRR) for transition to death.

For the age variable, we employed natural splines with knots placed at empirically derived points based on distribution of age at transitions between morbidity states. Specifically, we first identified all transitions between LTC states in our dataset and extracted the ages at which these transitions happened. We then calculated quantiles (5th through 95th percentiles) of the age-at-transition distribution. The natural spline transformation with these data-derived knots (52, 63, 69, 73, and 79 years) allowed for flexible modeling of age effects on transition hazards. Time since enrollment was modeled similarly, using natural splines with knots at 1, 5, 10, and 20 years. In addition, the current number of LTCs, representing the individual’s time-varying disease burden, was included as a covariate in all models. This allowed transition rates to be conditioned on the existing morbidity state at each time point. The base model (Model 0) additionally adjusted for calendar time (centered at 2004), sex, and ethnicity. We then added SES indicators (education, family income, TDI, and IMD) to these baseline factors (Model 1). Models were analyzed separately for each SES indicator. We then extended the model by including lifestyle score and BMI (Model 2) to investigate additional risk factors influencing LTC progression. BMI was modeled as a continuous variable using natural splines with knots at clinically relevant thresholds (25, 27, and 30 kg/m²). Models 1 and 2 assumed a proportional association between SES and morbidity transitions. In Model 3, interaction terms between the current number of LTCs and each SES indicator were introduced, allowing transition-specific SES associations and thereby assessing how associations with SES varied across LTC states.

Finally, we visualized LTCs progression across different SES groups by generating predicted transition rates from our fitted multistate models. Using age as the underlying time scale we created visualizations for three age ranges (55–70 years, 60–75 years, and 65–80 years). For each age range and SES group, we applied the estimated models of standardized analytical profiles (e.g., white male, at 2018-1-1) to predict transition probabilities for both disease accumulation (transitions to subsequent LTC states) and mortality, comparing the highest versus lowest SES groups. From these we derived transition probability matrices at 0.1 year intervals (~1.2 months), using these to compute the probability of being in different states by age, allowing a comprehensive visualization of how individuals with different baseline characteristics through states of morbidity and mortality.

### Subgroup analyses

To examine whether results were consistent across sex, we conducted sex-stratified analyses. Separate multistate models were fitted for men and women, maintaining identical model specifications and covariate adjustments as the primary analysis.

All analyses were conducted in R (version 4.4.0). The multi-state models were constructed using the “*Epi*” and “*popEpi*” packages in R^30^.

### Ethics approval and consent to participate

The UK Biobank study received ethical approval from the North West Multi-centre Research Ethics Committee (MREC reference: 11/NW/03820). All participants gave written informed consent before enrolment in the study, which was conducted in accordance with the principles of the Declaration of Helsinki. Secondary analysis of UK Biobank data does not require additional ethical approval, as this is covered under the original UK Biobank ethics framework. This study has been conducted using the UK Biobank Resource under Application Number 81520.

## Results

### Descriptive data

The whole UKB cohort included 502,368 participants at baseline. 273,301 (54%) were female, the mean age was 56.5 (SD = 8.1) years, and a majority was white (94.4%). At baseline, 40.6% of college graduates had no chronic conditions, while only 22.8% had ≥4 LTCs. In contrast, among those with no qualifications, only 9.1% had no conditions, while 27.2% had ≥4 LTCs. Income showed similar disparities: in the highest income group (>£100,000), 7.3% had no conditions and 1.9% had ≥4 LTCs, compared to the lowest income group (<£18,000) where 11.7% had no conditions and 29.5% had ≥4 LTCs. Similar socioeconomic gradients were observed using area-based measures (TDI and IMD). 34.4% of the participants were former smokers, and 10.5% were current smokers. 3.6% of the participants were previous drinkers and 91.6% were current drinkers. 42.2% of the participants had BMI 25–29.9 kg/m² and 24.3% had BMI ≥ 30 kg/m². After 15.8 years of follow-up (IQR: 14.6–16.1), 26% (*n* = 130,429) remained free of LTCs, 20% (*n* = 101,948) had 1 LTC, 14% (*n* = 72,473) had 2 LTCs, 11% (*n* = 54,360) had 3LTCs, and 29% (*n* = 143,158) had ≥4 LTCs (Supplementary Data 4).

### Description of LTCs progression

**Fig. 1:**
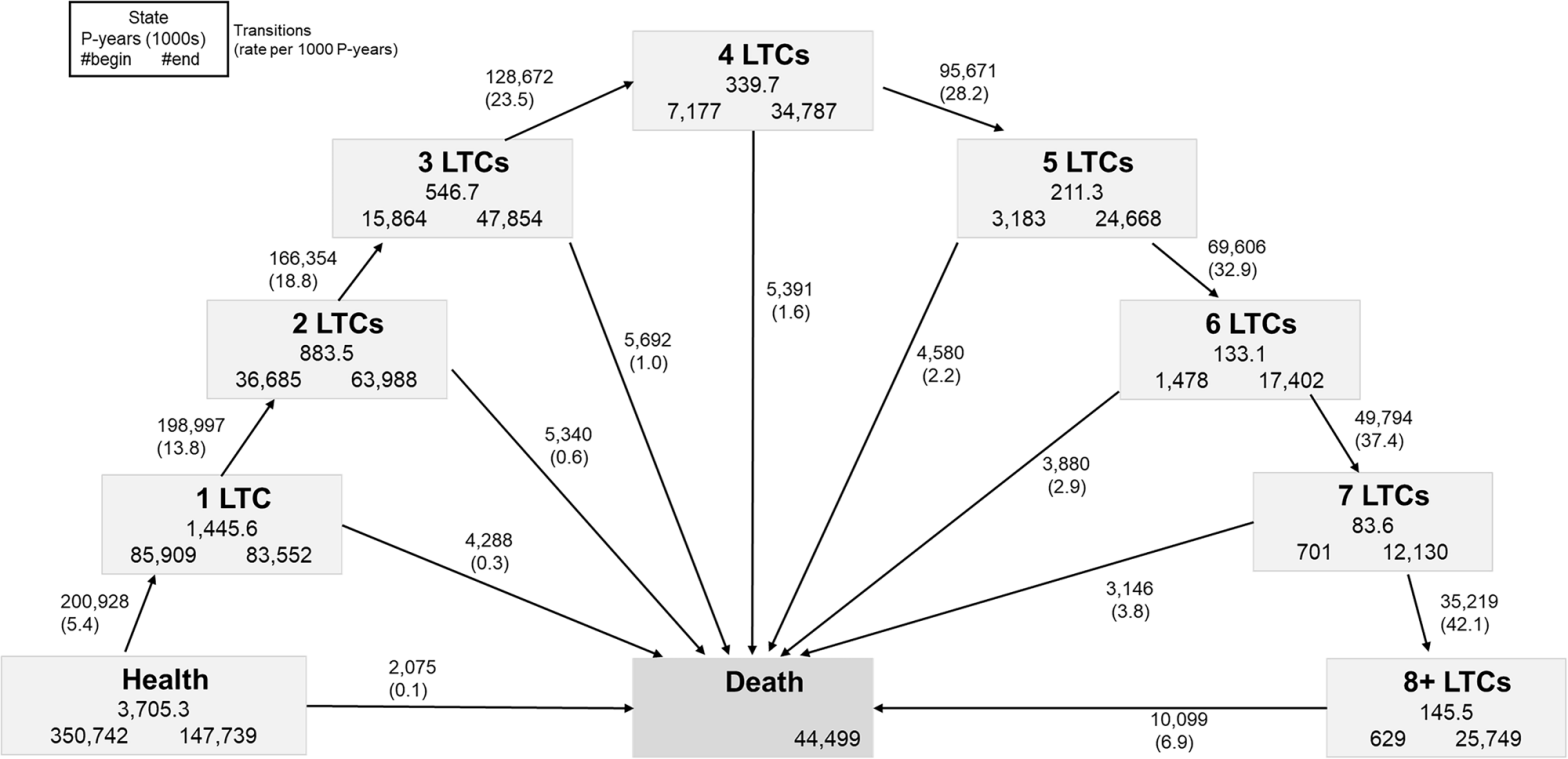
Multimorbidity and mortality in a multistate Markov model for all participants. The model assumes a progressive process in which individuals can only gain a single LTC at a time and cannot revert to a previous state, with death as the absorbing (final) state. Numbers at the bottom of each box denote the number of people who begin, respectively, end follow-up in that state. Transitions between states are indicated by arrows. Numbers by arrows are the number of transitions and numbers in brackets are transition rates per 1000 person-years of follow-up. *n* = 502,368 individuals contributing 7.5 million person-years of follow-up across all morbidity transitions. LTC long-term conditions.

A total of 502,368 participants were followed for 7.5 million person-years (PY). Figure 1 illustrates the crude transition rates to subsequent LTC stages. The transition rate increased with each subsequent LTC: from health to the first LTC (5.4 per 1000 PY), from 1 to 2 LTCs (13.8), from 2 to 3 LTCs (18.8), from 3 to 4 LTCs (23.5), and reached 42.1 per 1000 PY for transitions from 7 to 8+ LTCs. Among 44,499 participants who had a recorded date of death during the observation period, mortality risk increased with the number of LTCs (Fig. 1).

The number of LTCs increased with age and calendar year for both men and women. The median baseline age for those with no conditions was 61 years. As conditions accumulated, age gaps became smaller between LTC stages: participants with 1 LTC had a median age of 66 years, those with 2 LTCs were 68 years old, and those with 4 LTCs had a median age of 71 years. This pattern was similar for both males and females (Supplementary Data 5).

The number of existing LTCs was strongly associated with an increased rate of developing additional conditions. Compared to the rate of developing the first condition (from 0 to 1 LTC), the rate of acquiring a second condition (from 1 to 2 LTCs) was 2.27 times higher (95% CI: 2.25–2.28). This increasing trend continued across successive transitions, with the TRR reaching 6.01 (95% CI: 5.94–6.09) for individuals developing an eighth condition (from 7 to 8+ LTCs). Additionally, older age, male sex, non-white ethnicity, and calendar years were all associated with higher rates of LTC accumulation (Model0, Supplementary Data 6).

The relative risk of mortality showed a steeper increase with each additional LTC. Compared to individuals with no LTCs of the same age, those with one LTC had 4.77 times (95% CI: 4.52-5.03) higher risk of death, increasing to 9.06 for 2 LTCs (95% CI: 8.61–9.54) and reaching 93.50-fold for individuals with 8+ LTCs (95% CI: 88.94–98.30) (Model0, Supplementary Data 6).

### Associations of SES with morbidity and mortality rates

**Fig. 2:**
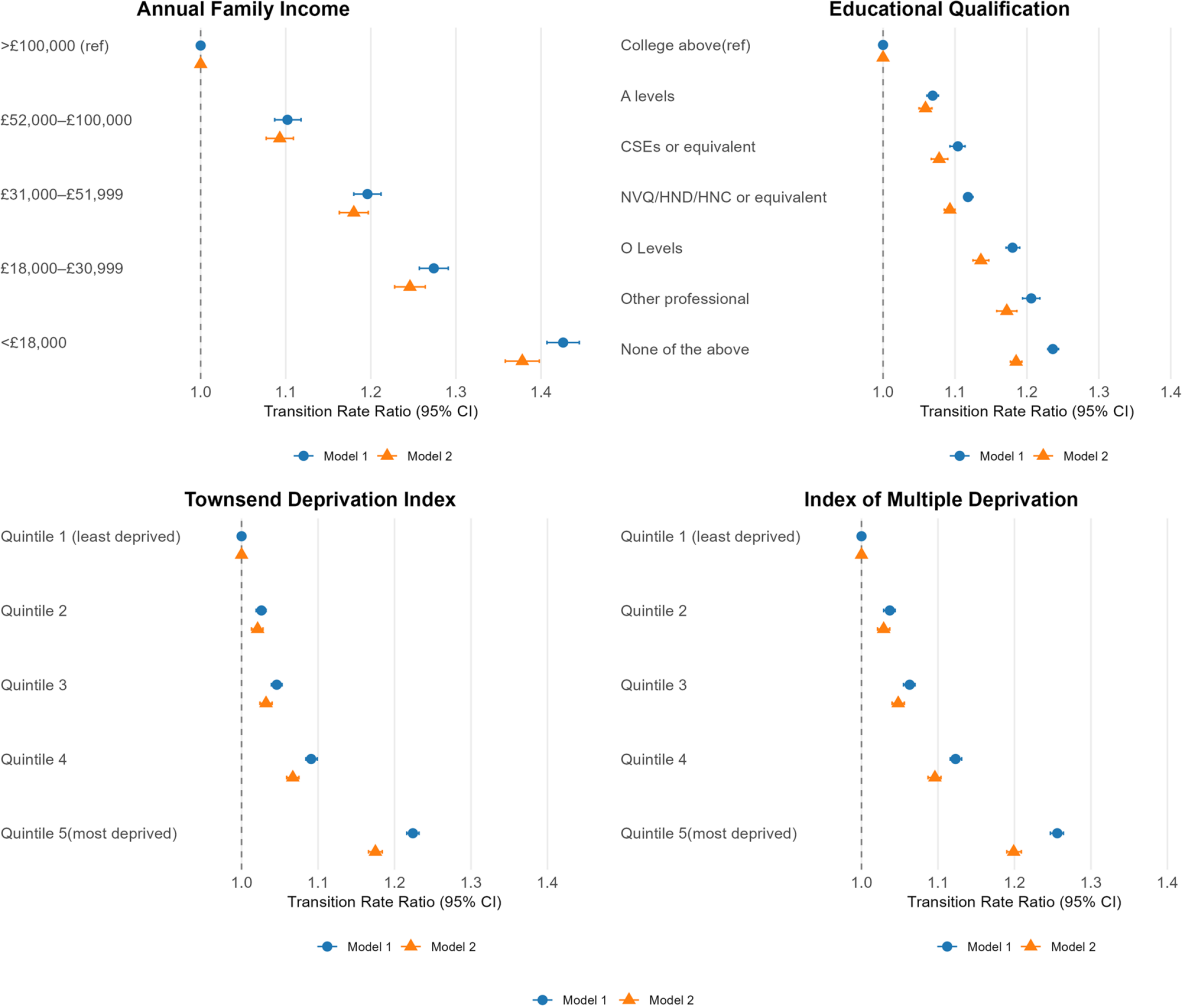
Average transition rate ratios of LTCs accumulation by socioeconomic status in multistate models. The figure shows the average transition rate ratios of LCT accumulation according to four socioeconomic status indicators. Models assume proportional socioeconomic status association across all transitions between morbidity states. Analyses were done by fitting separate multistate models for family income, education qualification, Townsend deprivation index, and index of multiple deprivation. Model 1 adjusted for age, time since enrollment, calendar year, sex, and ethnicity (blue dots); Model 2 further adjusted for lifestyle score and body mass index (orange triangles). Markers represent the estimated transition rate ratio (point estimate), with horizontal error bars indicating the corresponding 95% confidence interval (point estimate, 95% CI). (*n* = 424,120 participants for income models, *n* = 490,592 for education models, *n* = 499,868 for Townsend deprivation index models, and *n* = 430,929 for index of multiple deprivation models). CI confidence interval, LTC long-term conditions, ref reference.

There was a gradient between family income and the overall rate of LTC accumulation across all transitions. In model 1, which assumes a proportional SES association across transition states, individuals with lower family income had higher risks of developing additional LTCs. Compared with those in the highest family income bracket (>£100,000), average TRRs were higher with lower income: £52,000–£100,000: TRR = 1.10 (95% CI: 1.09–1.12), £31,000–£51,999: TRR = 1.19 (95% CI: 1.18–1.21), £18,000–£30,999: TRR = 1.27 (95% CI: 1.26–1.29), <£18,000: TRR = 1.43 (95% CI: 1.41–1.45) (Fig. 2). After adjusting for the summed lifestyle factor score and BMI, the associations between family income and the rate of LTC accumulation were slightly attenuated but remained significant (Model 2). Similarly, family income was associated with mortality risk, though the magnitude of the gradient was smaller, indicating that mortality is mostly driven by the number of chronic conditions rather than income directly. Individuals in the lowest income group (<£18,000) had a 1.24 times higher risk of death (MRR = 1.24, 95% CI: 1.15–1.34) compared to those in the highest income group (Model2, Supplementary Data 7).

Educational attainment followed a similar gradient pattern, where individuals with lower education levels had higher overall rates of LTC accumulation compared to those with college education or above. The highest morbidity risk was observed in individuals with no qualifications (TRR = 1.24, 95% CI: 1.23–1.24), followed by CSEs (TRR = 1.21, 95% CI:1.19–1.22), NVQ/HND/HNC (TRR = 1.18, 95% CI:1.17–1.19), O levels (TRR = 1.12, 95% CI:1.11–1.13), other professional qualifications (TRR = 1.10, 95% CI:1.09–1.11), and A levels (TRR = 1.07, 95% CI:1.06–1.08) (Fig. 2). Education was not associated with mortality risk, except among individuals with no formal qualifications, who had a slightly higher risk of mortality compared to those with higher education (MRR = 1.07, 95% CI: 1.04–1.11) (Model2, Supplementary Data 8).

Area-based deprivation measures showed consistent inverse associations with rate of LTC accumulation. Compared to individuals in the least deprived quintile, those in the most deprived quintile had higher risks of morbidity progression, with TRRs ranging from 1.03 to 1.22 across TDI quintiles and 1.04 to 1.26 across IMD quintiles (Fig. 2). Both TDI and IMD were associated with greater mortality risk with similar magnitudes (Supplementary Data 9,10).

### Transition-specific Analysis of SES and Morbidity Transitions

**Fig. 3:**
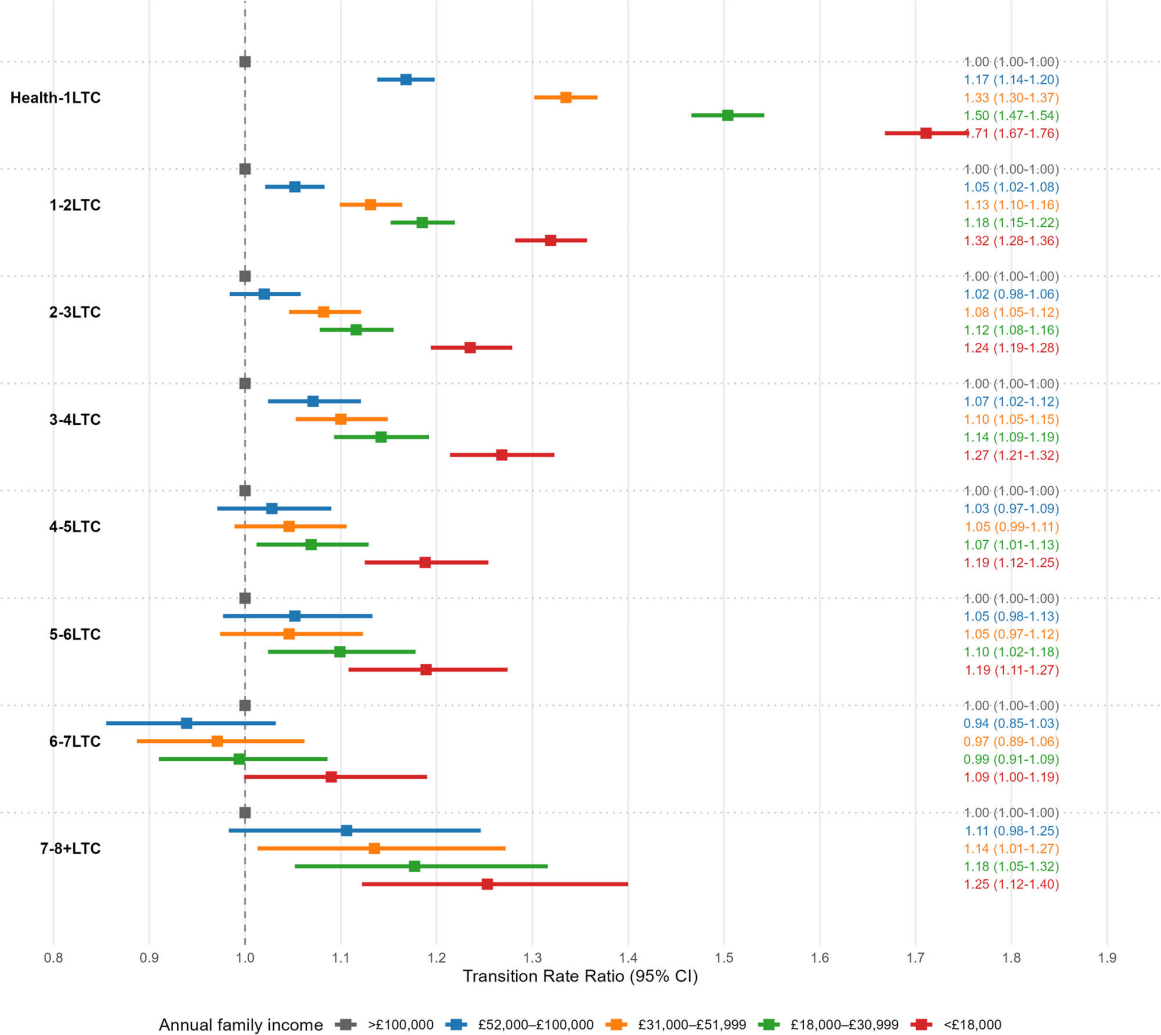
Transition-specific rate ratios for LTCs accumulation by annual family income. The figure shows transition rate ratios for LTC accumulation by family income at each transition stage, from health to 1 LTC, 1 to 2 LTCs, and sequentially up to 7 to 8+ LTCs. Model 3 allowed for transition-specific socioeconomic associations by including interaction terms between the current number of LTCs and income, thereby permitting income–LTC associations to vary across LTC states. Markers represent the model-estimated transition rate ratio (point estimate), with horizontal error bars indicating the corresponding 95% confidence interval (point estimate, 95% CI). (*n* = 424,120 participants for family income models). Reference group: family income >£100,000. LTC Long-term conditions, CI Confidence intervals, ref reference.

In transition-specific analyses, family income was inversely associated with accumulation rate of additional LTCs across each transition state, though the magnitudes of association varied (Model 3, Supplementary Data 11). Family income demonstrated a strong association with the transition from 0 (health) to the first LTC, with individuals in the lowest income category (<£18,000) having a 1.71-fold greater transition rate (95% CI: 1.67–1.76) compared to those in the highest income level (>£100,000). This association weakened in subsequent transitions, with the TRRs decreasing to 1.32 (95% CI: 1.28–1.36) for progression from 1 to 2 LTCs and remaining around 1.2 for later transitions (Fig. 3, Supplementary Data 11).

Education was consistently inversely associated with LTC accumulation rates across transition states. Lower education showed a stronger association for the transition from 0 (health) to 1 LTC, exhibiting a TRR of 1.44 (95% CI: 1.42–1.46) for those with no education compared with those with a college degree. The associations were lower at 1.20 (95% CI: 1.18–1.22) for the transition from 1 to 2 LTCs, and further at 1.12 (95% CI: 1.10–1.14) from 2 to 3 LTCs, then remained around 1.1 for the following transition states. The other levels of education demonstrated the same patterns, showing higher TRRs for the initial transition, which then stabilized in the following transition states, though at comparably lower magnitudes (Supplementary Fig. S1, Supplementary Data 11).

**Fig. 4:**
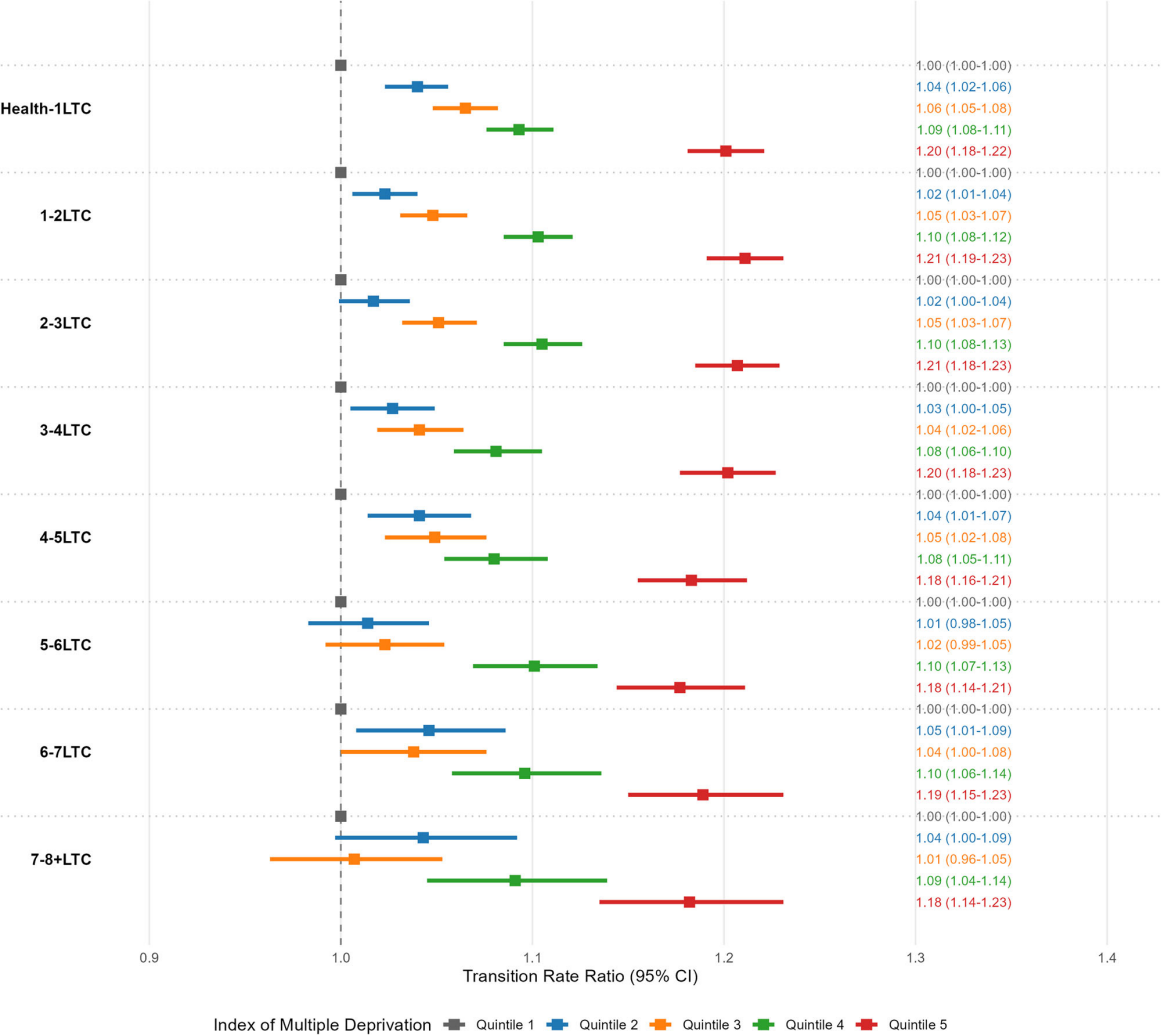
Transition-specific rate ratios for LTCs accumulation by index of multiple deprivation. The figure shows transition rate ratios for LTC accumulation by IMD at each transition stage, from health to 1 LTC, 1 to 2 LTCs, and sequentially up to 7 to 8+ LTCs. Model 3 allowed for transition-specific socioeconomic associations by including interaction terms between the current number of LTCs and IMD, thereby permitting IMD–LTC associations to vary across LTC states. Markers represent the model-estimated transition rate ratio (point estimate), with horizontal error bars indicating the corresponding 95% confidence interval (point estimate, 95% CI). (*n* = 430,929 participants for IMD models). Reference group: Quintile 1 (least deprived). LTC Long-term conditions, CI Confidence intervals, ref reference.

Residents in the most deprived areas had higher transition rates compared to those in the least deprived areas (IMD) across all stages, with TRRs staying around 1.2 across transitions (Fig. 4, Supplementary Data 11). TDI showed consistent associations with LTCs accumulation rates but exhibited stronger magnitudes at later stages. The TRRs ranged from 1.12 (95% CI: 1.10–1.14) to 1.21 (95% CI: 1.16–1.26) from stage 0-1 LTCs to 7-8+ LTCs when comparing the most deprived to the least deprived group (Supplementary Fig. S2, Supplementary Data 11). A similar pattern between SES and mortality rates was observed, with wider CIs.

We plotted the state probability of LTC accumulation and mortality rates by age. Individuals with the highest family income maintained healthier states (fewer LTCS) longer, experienced delayed accumulation of additional LTCs, and had a lower probability of death compared to those with the lowest family income. The income-based disparity was substantial. For example, individuals with family income greater than £100,000 reached a 20% probability of having at least 2 LTCs 2.3 years later than those with income less than £18,000, and reached a 20% probability of having at least 3 LTCs 3.6 years later (predicted age range: 60–75; Fig. 5).

**Fig. 5:**
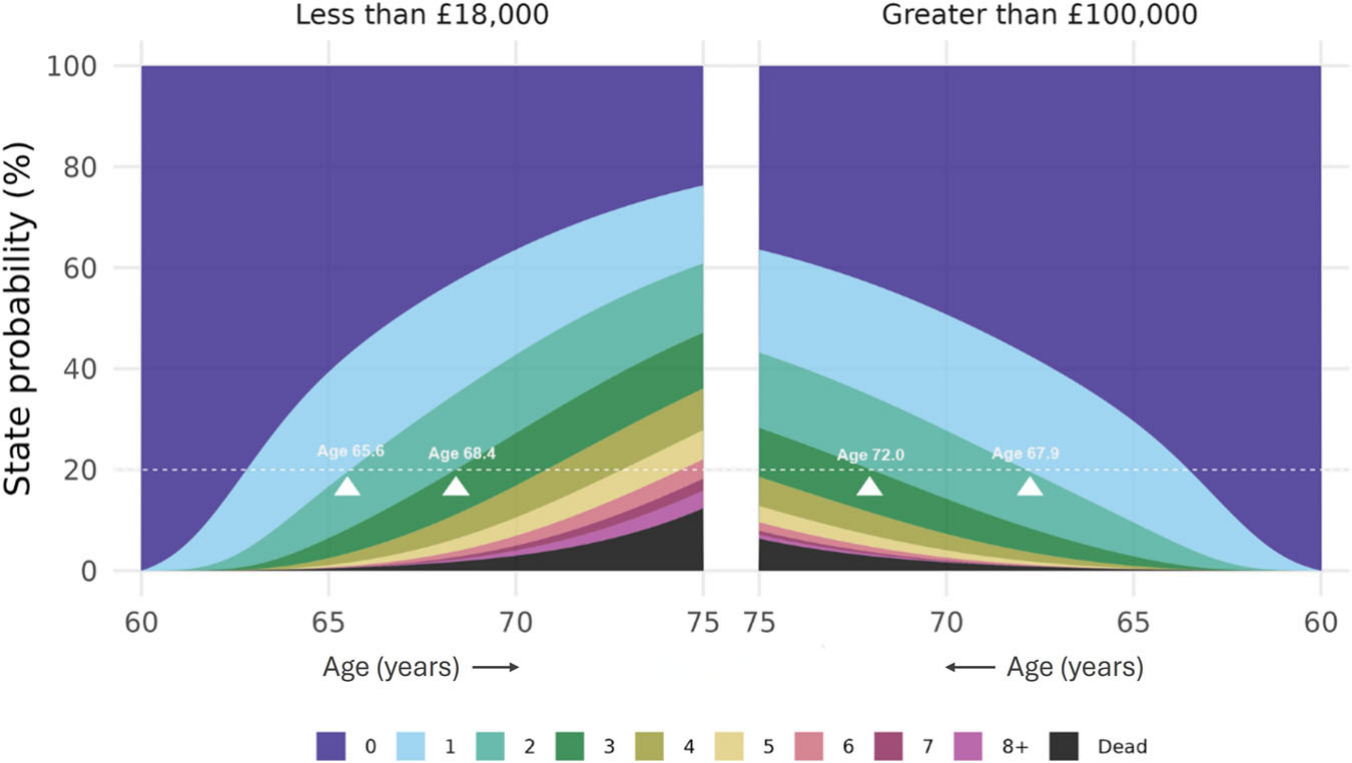
Predicted state probabilities of LTC accumulation and mortality by family income. Left panel: lowest family income (<£18,000); right panel: highest family income (>£100,000). Note that the right panel x-axis is reversed to facilitate mirrored comparison between income groups; both panels cover ages 60–75. Each colored area represents the probability of being in a given morbidity state at a given age for persons [Male, White, 2018] entering at age 60. Boundaries between adjacent areas show probabilities at least a given number of LTCs (e.g. between ‘1’ and ‘2’ shows P(≥2LTCs)). Where the horizontal dashed line at 20% intersects the boundaries mark the ages at which one-fifth of the population reaches each morbidity threshold. Differences in age at 20% probability between income groups indicate income disparities in disease accumulation. For example, individuals with family income greater than £100,000 reached a 20% probability of having at least 2 LTCs 2.3 years later than those with income less than £18,000, and reached a 20% probability of having at least 3 LTCs 3.6 years later. LTC Long-term conditions.

The health disparities in LTC accumulation were observed across all SES indicators with individuals of advantaged SES experiencing relatively lower risks of progression from a single condition to multimorbidity and death, compared to their less affluent counterparts across all transition stages (Supplementary Data 12, Supplementary Figs. S3–S6).

The predicted transition rates of LTC accumulation and mortality rates increased exponentially with age across all LTCs states. Individuals with lower SES experienced elevated transition rates to additional LTCs compared to their more affluent counterparts, with differences evident across all transition stages. In parallel, socioeconomic gradients in mortality were observed within each LTCs, persisting even among individuals with high disease burden (Supplementary Figs. S7–S10).

### Subgroup analyses

When examining SES across sex-stratified analyses, both men and women showed comparable socioeconomic gradients in multimorbidity progression rates (Supplementary Data 13–16). The magnitude of SES associations with LTC accumulation was similar between sexes.

## Discussion

In this population-based cohort study with 7.5 million person-years of follow-up, we found that socioeconomic disadvantage was consistently associated with accelerated development of multiple chronic conditions. Individuals with lower SES experienced substantially higher rates of LTCs accumulation and mortality, which persisted even after accounting for lifestyle factors. The SES association varied by the transition states, with stronger associations observed in early disease transitions for family income and education. These findings demonstrate that SES influences both the onset of chronic disease and the rate at which additional conditions are acquired.

We analyzed occurrence rates of disease accumulation across 80 LTCs and demonstrated that individuals from disadvantaged SES followed a faster pattern of disease accumulation compared to those with advantaged status. A consistent gradient association was observed between all four SES indicators (family income, education, TDI, IMD) and higher rates of LTCs accumulation. Furthermore, we extended previous research by examining transition-specific rates from health to 8+ LTCs. Socioeconomic indicators, particularly family income and education, showed varying associations across different stages of disease accumulation. The strongest associations were observed in the transition from healthy status to the first chronic condition, with magnitudes diminishing and stabilizing in subsequent transitions. Our results are consistent with a study using Whitehall II data with 23.7-year mean follow-up, which reported that adverse midlife occupational position was associated with higher risk of disease progression: 42% increased risk (HR: 1.42; 95% CI: 1.23–1.64) for developing the first cardiometabolic disease, and 54% increased risk (HR: 1.54; 95% CI: 1.10–2.15) for progressing from one to cardiometabolic multimorbidity^31^. These findings suggest that individual SES is especially critical in disease initiation and early multimorbidity development, highlighting the importance of early preventive interventions. We also observed that the strengths of associations between area deprivation and morbidity showed a consistent pattern across transitions, with TRR around 1.2 for IMD and 1.1 for TDI. This strength of association was a bit lower than a retrospective cohort conducted in the UK using primary care data, reporting the TRR between deprivation and transition from one to two LTCs was between 1.30 and 1.64^11^. The stronger association in the later study may be explained by its use of primary care data from a more deprived population. Nevertheless, the persistent association of area-level deprivation across all transition stages, albeit of moderate magnitude, suggests that neighborhood environment exerts a continual influence throughout the entire disease accumulation journey.

The more pronounced link between individual-level SES (particularly family income) and initial disease onset suggests that personal material resources play a role in the earliest stages of chronic disease development. Higher income in adulthood may reflect adequate family wealth and better access to opportunities earlier in life, enabling individuals to engage in primary prevention through better nutrition, physical activity, stress management, and early screening^32–34^. These mechanisms may be more relevant before the first chronic condition develops^35^. Once an individual develops one or more conditions, the marginal effect of individual resources for preventing additional conditions may diminish, as managing existing conditions become more complex and may require resources beyond what income alone can provide. Conversely, the persistent influence of area-level deprivation across all stages of disease accumulation suggests that neighborhood contextual factors create enduring structural barrier that affect disease accumulation throughout the entire trajectory^36^. Deprived areas create persistent structural barriers, such as poor continuity of care or reduced access to integrated multidisciplinary service^37^. Additionally, environmental exposures common in deprived areas (air pollution, noise, lack of green space) may contribute to chronic inflammation and allostatic load that continuously drives disease progression^38^. These patterns are consistent with the social-ecological framework^39^ that different ecological levels exert varying influence on different health states, suggesting that while individual resources are crucial for primary prevention, contextual factors are salient for managing existing conditions and preventing further accumulation.

Our results confirm the importance of SES as one root cause of multimorbidity, highlighting the importance of addressing these upstream determinants of health. These findings align with the ‘common soil’ hypothesis^40,41^, which suggests that socioeconomic disadvantage may compromise physiological homeostasis through chronic stress exposure and elevated inflammation or infection, creating a physiological environment vulnerable to disease development and progression of multiple LTCs^42^. The attenuation of associations we observed upon adjustment for health behavioral covariates implies that shared lifestyle factors explain part, but not all, of the SES-LTC gradient. Significant associations between lower SES and faster disease accumulation rates persisted even after these adjustments, indicating that SES may influence multimorbidity through additional biological and structural pathways beyond health behaviors^43^. It should be noted that using a composite measure for health behaviors may oversimplify individual behaviors and be subject to residual confounding. In addition, the interpretation of health outcomes by SES should be cautious. Income is less of a robust SES measure particularly among retired people. There might be bidirectional relationship between family income and disease accumulation since income was assessed only at baseline. Due to data limitations, we only examined SES as a baseline determinant and could not examine potential changes in SES over time. Future studies incorporating longitudinal SES measures could provide deeper insights into the dynamic relationship between SES and disease onset and the subsequent acquisition of additional conditions.

We also observed that once individuals develop their first condition, they often progress more rapidly to subsequent conditions. The strong association between existing and subsequent conditions could be explained by both direct and indirect pathways. Direct pathways involve pathophysiological processes where one condition triggers or accelerates others, particularly through immune response and inflammation^44^. Indirect pathways include closer clinical attention and faster diagnostic cycles. Aging itself reduces physiological resilience and heightens vulnerability to multiple interacting conditions, creating a biological foundation upon which both direct and indirect pathways operate more efficiently. Multimorbidity is of course also linked to increased pharmacological treatment and to responsive lifestyle modifications, which could in turn impact the risk of further morbidity and mortality. For example, individuals with mobility-limiting conditions like severe arthritis may become less physically active, leading to weight gain and increased risk of developing conditions such as diabetes and cardiovascular disease, thus accelerating their progression of multimorbidity^40^.

A key strength of this study is that we used population-based data with comprehensive morbidity tracking to examine disease accumulation patterns. Using multistate models, we could simultaneously analyze all forward transitions up to 8+ LTCs, extending previous work limited to 3 + LTCs^11^. LTCs were objectively measured from hospital records, ensuring greater accuracy and reducing self-reporting bias. Secondly, we measured SES using four distinct indicators (education, family income, TDI, and IMD), all of which demonstrated similar and moderate association of disease onset and accumulation rate. Importantly, while these inverse associations between SES and disease accumulation varied in magnitude across different transition stages, they remained statistically significant throughout the entire multimorbidity journey, including later stages. These findings highlight the complex relationship between socioeconomic disparity and health outcomes, suggesting that comprehensive approaches addressing both behavioral and structural determinants are necessary to reduce disparities in multimorbidity development^45^.

Our study has some limitations. Firstly, we chose to focus on the onset of any condition from our predefined list instead of examining specific diseases. While different chronic diseases have distinct associations with SES and mortality, our primary aim was to quantify how SES influences the overall rate of LTCs accumulation as a proxy of disease burden. We counted only the first occurrence of a condition within each disease category, an approach that aligns with our focus on examining LTC accumulation rather than tracking concisely defined current states. This “forward-only” framework amounts to an assumption that the cumulative number of LTCs provides an adequate account of disease burden. Future work with more complete remission data could examine whether accounting for disease resolution alters SES disparities in multimorbidity progression. Secondly, our reliance on hospital admissions and outpatient consultation data may underestimate the true incidence of multimorbidity, particularly for mental health conditions and for other conditions treated exclusively in primary care. The use of hospital records may also introduce detection bias, as individuals with an existing condition may have more frequent healthcare contacts, leading to increased opportunities for diagnosing additional conditions. This bias could artificially inflate the speed of multimorbidity progression among those with an initial condition. However, the purpose of the study is to compare the transition rates between SES groups. Therefore, even if the overall progression speed was overestimated, the relative differences between SES groups are unlikely to be materially affected. Thirdly, disease onset dates in electronic health records may not always reflect the true clinical onset, and some same-day diagnoses likely represent coding practices rather than truly simultaneous disease events (Supplementary Data 17, 18). Our model assumes that no “simultaneous events” occurred on the same day, which may slightly underestimate transition rates for highly correlated or clustered diseases. This limitation is partly due to data resolution rather than a purely methodological choice. Future research employing more advanced models (e.g., multivariate multistate models) could yield more clinically realistic estimates of disease progression. Lastly, the participants in UKB are known to be healthier and more educated than the general population^45^, which may have led to a relative underrepresentation of the disadvantaged SES groups, and of the least healthy within each group. If anything this selection might have led to our study underestimating the magnitude of true SES gradients in the general UK population. These considerations need to be taken into account when generalizing our findings to other populations.

## Conclusion

Our findings indicate that the influence of SES does not cease once individuals became morbid, but continues to shape the disease accumulation rate at each subsequent stages. Different measures of SES showed consistent associations with disease accumulation rate and mortality. People from disadvantaged SES experienced accelerated development of multiple chronic conditions, with income showing particularly strong effects - lowest income groups progressed to subsequent conditions up to 4 years earlier than highest income groups. These findings emphasize that SES is a critical risk factor for both disease onset and progression, highlighting the importance of addressing social inequalities to delay the accumulation of LTCs.

## Supplementary information

Supplementary Information

Description of Additional Supplementary files

Supplementary data1-18

## Data Availability

UK Biobank data are available to researchers through the standard application process (https://www.ukbiobank.ac.uk/enable-your-research/apply-for-access). This study was conducted under UK Biobank Application Number 81520. Source data underlying all figures and tables are provided in Supplementary Data (1-18). Additional materials are available from the corresponding author upon reasonable request.

## Code availability

The analysis code for this study has been deposited in the Zenodo repository (10.5281/zenodo.19183927)^46^ and is also available on GitHub at https://github.com/steno-aarhus/sesmm.
