# Supplementary material for: Relationship between socioeconomic inequality and multimorbidity progression in UK Biobank data
Source: Commun Med (Lond). 2026 May 5;6:387. doi: 10.1038/s43856-026-01607-5 (PMC13346834; doi:10.1038/s43856-026-01607-5)
Supplement: Supplementary file 1 — Supplementary Information [file 43856_2026_1607_MOESM1_ESM.pdf]

Supplementary Material File

Supplementary Figures

Figure S1. Transition-specific rate ratios for LTC accumulation by educational qualification
Figure S2. Transition-specific rate ratios for LTC accumulation by Townsend Deprivation Index
Figure S3. Predicted state probabilities of LTC accumulation and mortality by family income
Figure S4. Predicted state probabilities of LTC accumulation and mortality by education level
Figure S5. Predicted state probabilities of LTC accumulation and mortality by Townsend Deprivation Index
Figure S6. Predicted state probabilities of LTC accumulation and mortality by Index of Multiple Deprivation
Figure S7. Predicted transition rates of LTC accumulation and morality by family income
Figure S8. Predicted transition rates of LTC accumulation and mortality by education
Figure S9. Predicted transition rates of LTC accumulation and morality by Townsend Deprivation Index
Figure S10. Predicted transition rates of LTC accumulation and morality by Index of Multiple Deprivation

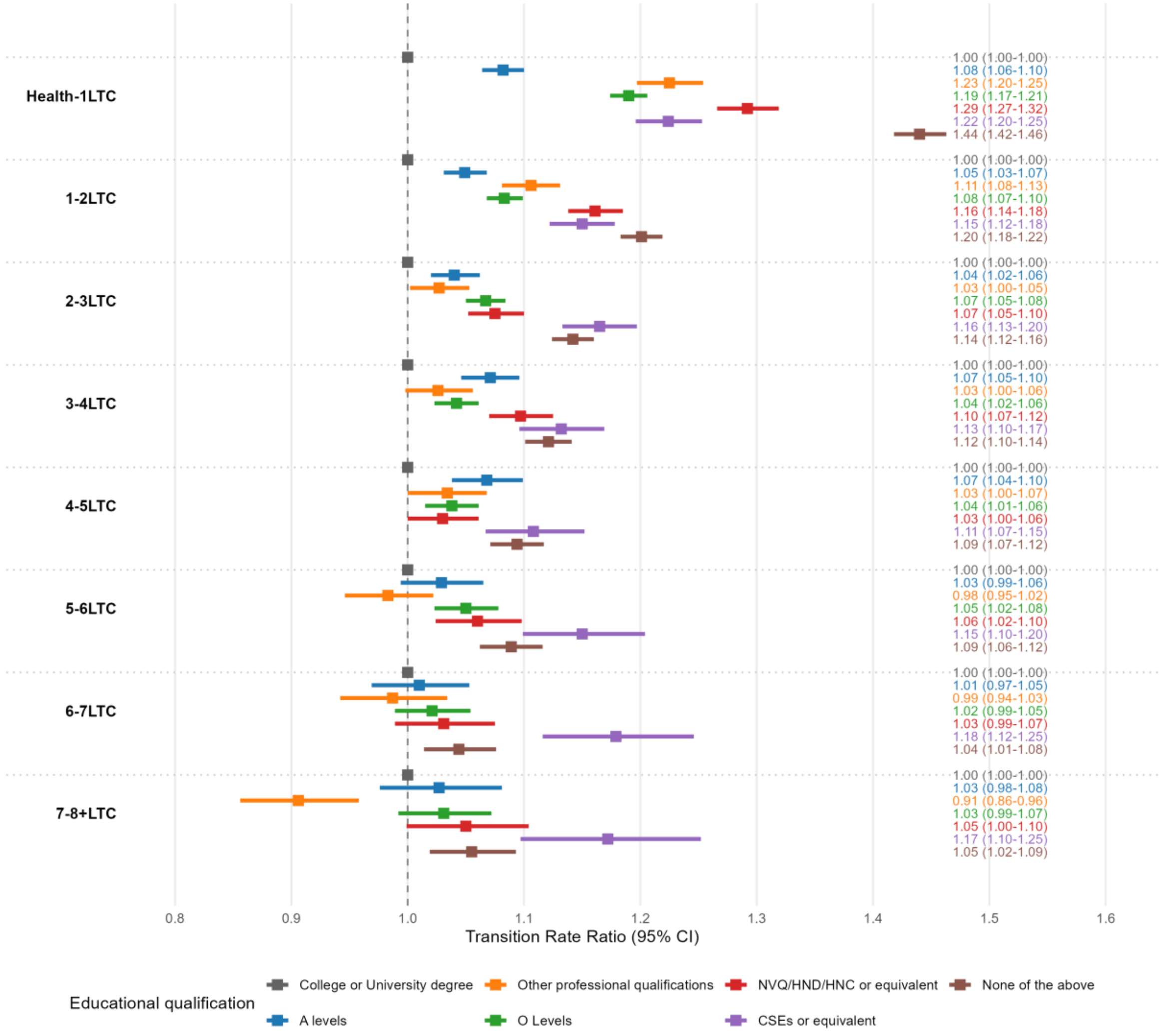

Figure S1. Transition-specific rate ratios for LTC accumulation by educational qualification

Abbreviations: LTC, Long-term conditions; CI, Confidence intervals. Reference group: college or university degree.

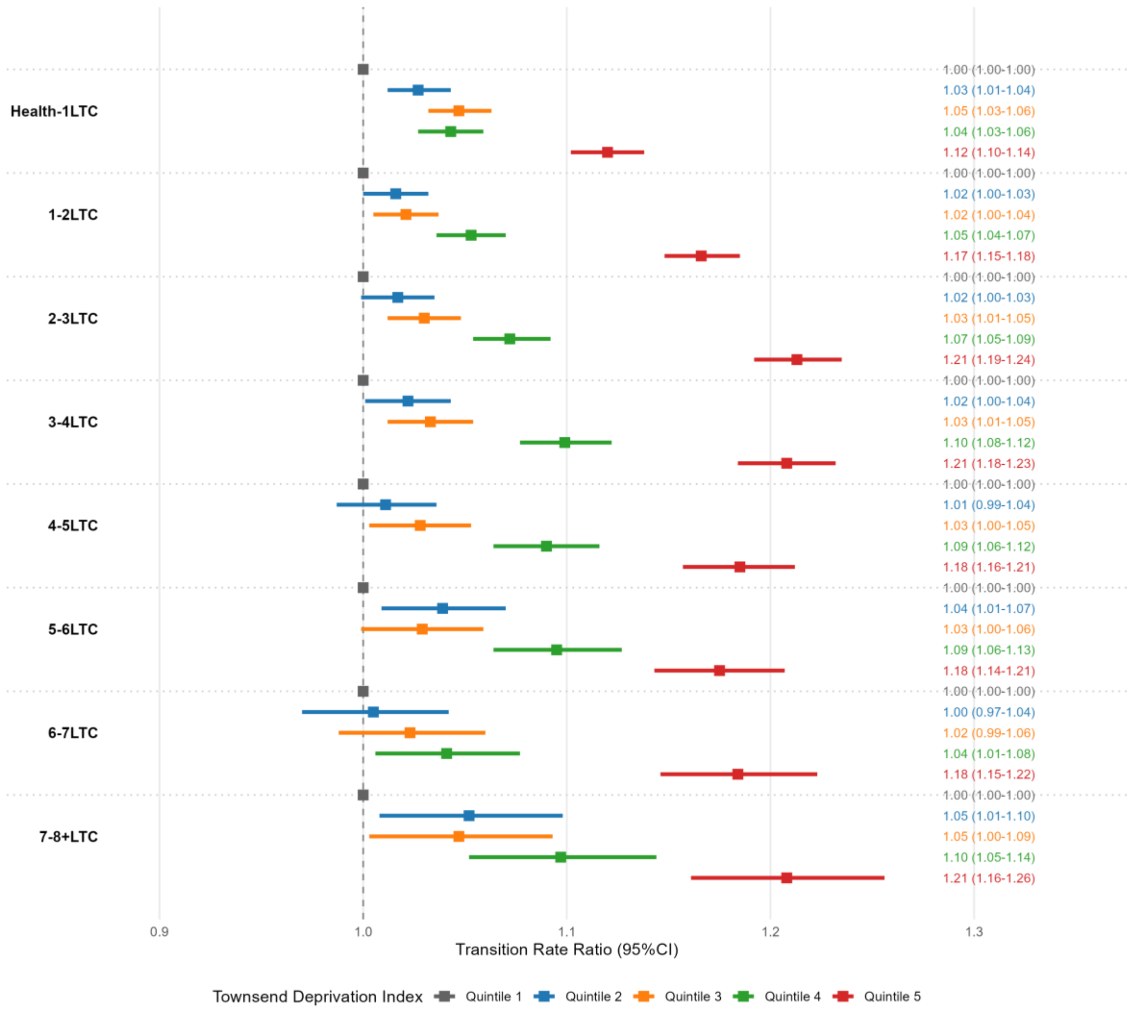

Figure S2. Transition-specific rate ratios for LTC accumulation by Townsend Deprivation Index

Abbreviations: LTC, Long-term conditions; CI, Confidence intervals. Reference group: Quintile 1 (least deprived)

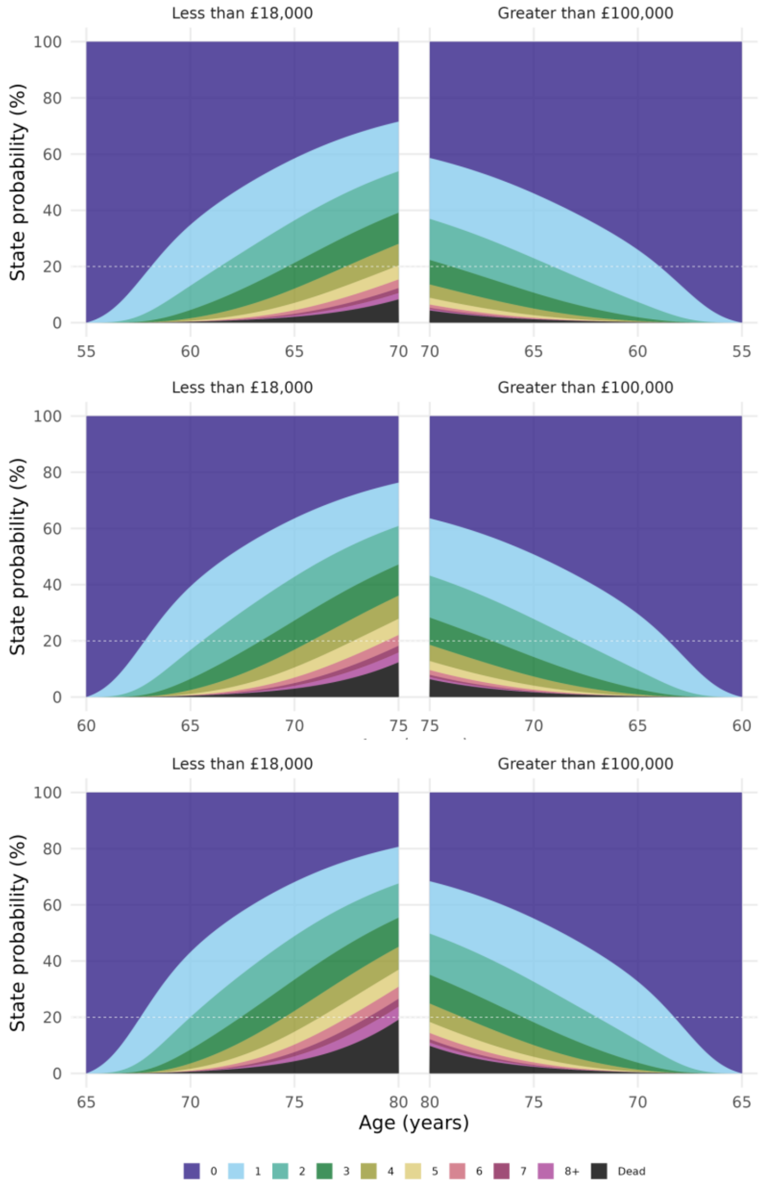

Less than £18,000
Greater than £100,000
State probability (%)
100
80
60
40
20
0
55
60
65
70
Less than £18,000
Greater than £100,000
State probability (%)
60
65
70
75
Less than £18,000
Greater than £100,000
State probability (%)
65
70
75
80
Age (years)
0
1
2
3
4
5
6
7
8+
Dead

Figure S3. Predicted state probabilities of LTC accumulation and mortality by family income

Predicted probabilities of having 0, 1, 2, 3, etc. LTCs. Each colored area represents the probability of being in a given morbidity state at a given age for persons [Male, White, 2018] entering at age 55, 60, or 65. Boundaries between adjacent areas show probabilities at least a given number of LTCs (e.g. between "1" and "2" shows P(⩾2LTCs)). Where the horizontal dashed line at 20% intersects the boundaries marks the ages at which one-fifth of the population reaches each morbidity threshold. Differences in age at 20% probability between income groups indicate income disparities in LTC accumulation.

Abbreviations: LTCs, long-term conditions.

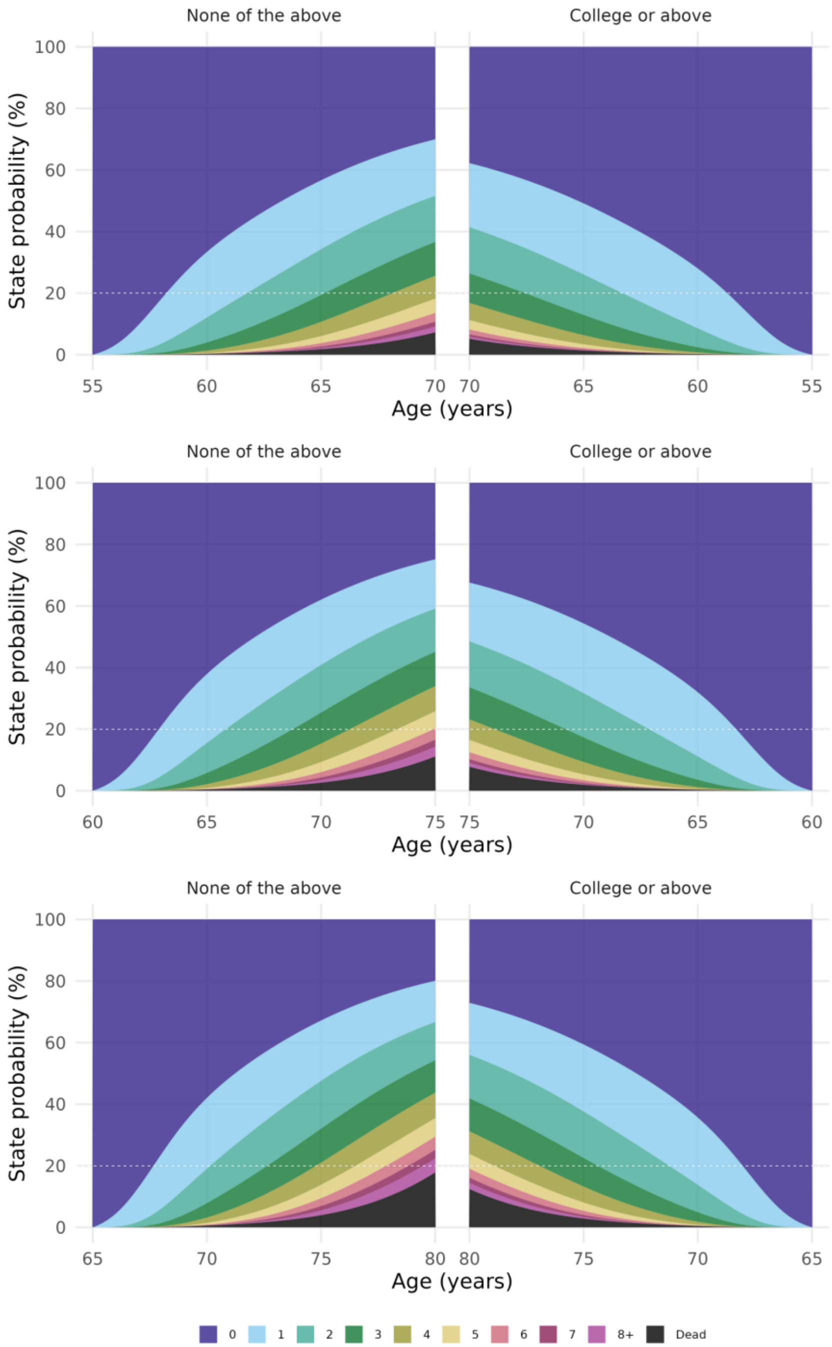
None of the above
College or above
State probability (%)
Age (years)
0
1
2
3
4
5
6
7
8+
Dead

Figure S4. Predicted state probabilities of LTC accumulation and mortality by education level

Predicted probabilities of having 0, 1, 2, 3, etc. LTCs. Each colored area represents the probability of being in a given morbidity state at a given age for persons [Male, White, 2018] entering at age 55, 60, or 65. Boundaries between adjacent areas shows probabilities at least a given number of LTCs (e.g. between "1" and "2" shows P(≥2LTCs)). Where the horizontal dashed line at 20% intersects the boundaries marks the ages at which one-fifth of the population reaches each morbidity threshold. Differences in age at 20% probability between education groups indicate education disparities in LTC accumulation.

Abbreviations: LTCs, long-term conditions.

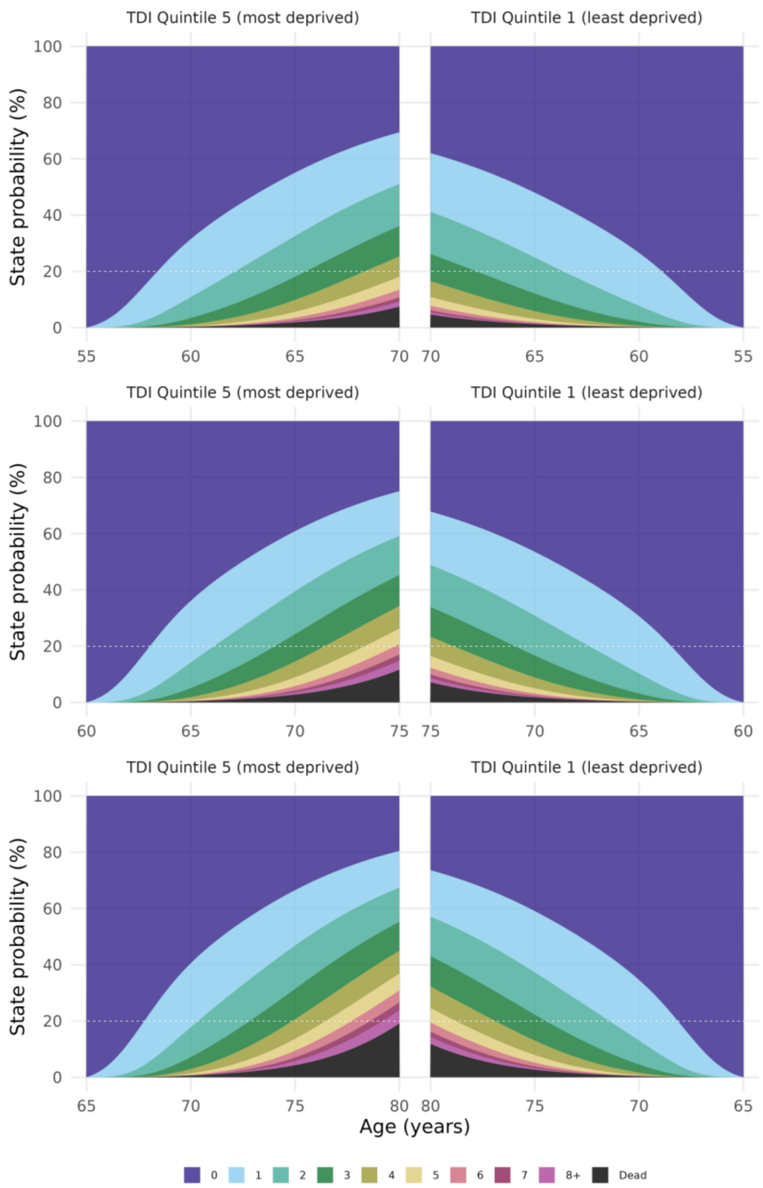
TDI Quintile 5 (most deprived)
TDI Quintile 1 (least deprived)
State probability (%)
100
80
60
40
20
0
55
60
65
70
TDI Quintile 5 (most deprived)
TDI Quintile 1 (least deprived)
State probability (%)
60
65
70
75
TDI Quintile 5 (most deprived)
TDI Quintile 1 (least deprived)
State probability (%)
65
70
75
80
Age (years)
0
1
2
3
4
5
6
7
8+
Dead

Figure S5. Predicted state probabilities of LTC accumulation and mortality by TDI

Predicted probabilities of having 0, 1, 2, 3, etc. LTCs. Each colored area represents the probability of being in a given morbidity state at a given age for persons [Male, White, 2018] entering at age 55, 60, or 65. Boundaries between adjacent areas shows probabilities at least a given number of LTCs (e.g. between "1" and "2" shows P($\geqslant$2LTCs)). Where the horizontal dashed line at 20% intersects the boundaries marks the ages at which one-fifth of the population reaches each morbidity threshold. Differences in age at 20% probability between TDI groups indicate disparities in LTC accumulation.

Abbreviations: LTCs, long-term conditions; TDI, Townsend deprivation index

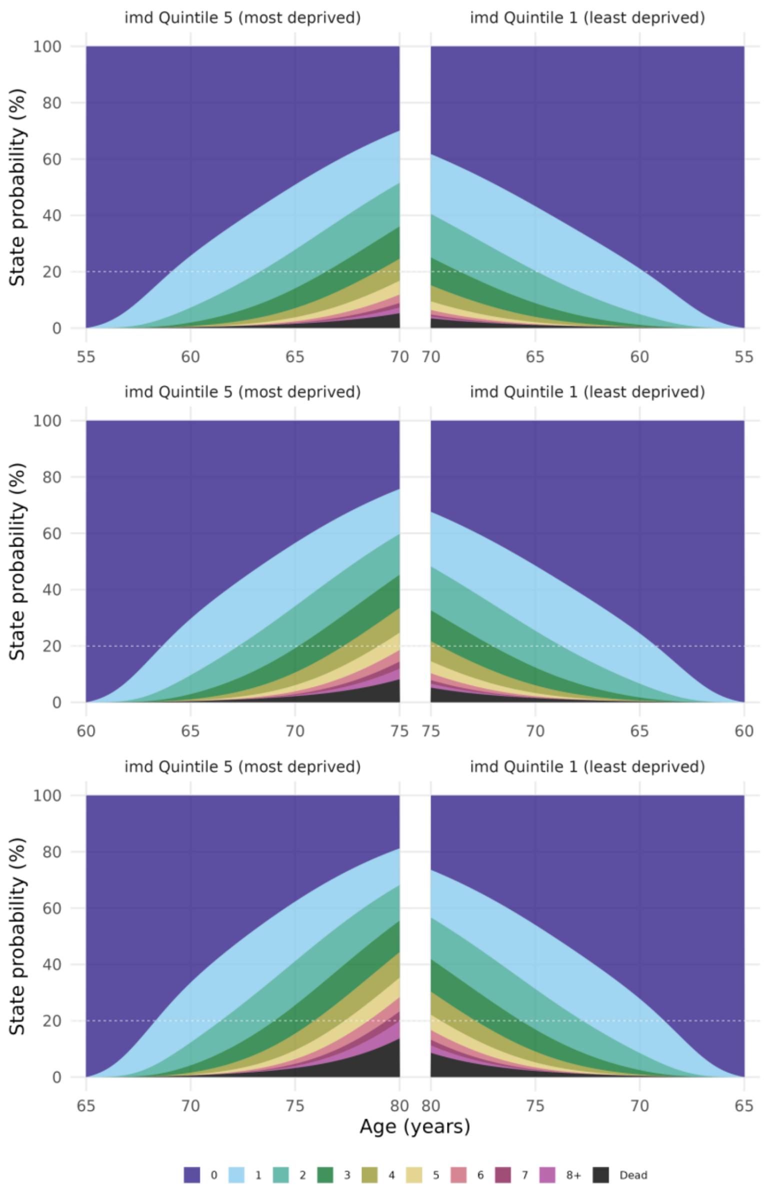
imd Quintile 5 (most deprived)
imd Quintile 1 (least deprived)
State probability (%)
Age (years)
0
1
2
3
4
5
6
7
8+
Dead

Figure S6. Predicted state probabilities of LTC accumulation and mortality by IMD

Predicted probabilities of having 0, 1, 2, 3, etc. LTCs. Each colored area represents the probability of being in a given morbidity state at a given age for persons [Male, White, 2018] entering at age 55, 60, or 65. Boundaries between adjacent areas shows probabilities at least a given number of LTCs (e.g. between "1" and "2" shows P(⩾2LTCs)). Where the horizontal dashed line at 20% intersects the boundaries marks the ages at which one-fifth of the population reaches each morbidity threshold. Differences in age at 20% probability between IMD groups indicate disparities in LTC accumulation.

Abbreviations:; LTCs, long-term conditions; IMD, index of material deprivation

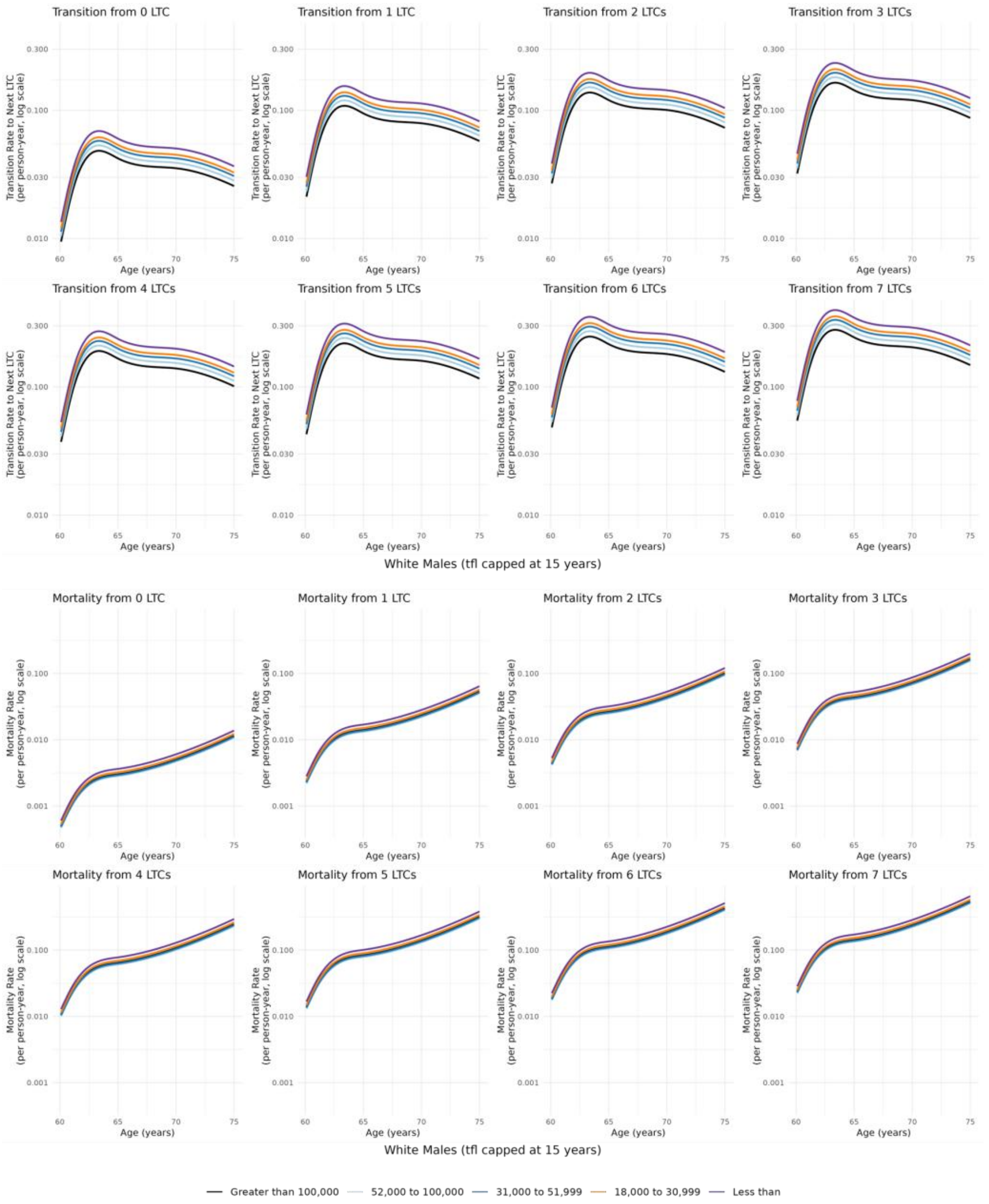
Transition from 0 LTC
Transition from 1 LTC
Transition from 2 LTCs
Transition from 3 LTCs
Transition from 4 LTCs
Transition from 5 LTCs
Transition from 6 LTCs
Transition from 7 LTCs
Transition Rate to Next LTC (per person-year, log scale)
Age (years)
White Males (tfl capped at 15 years)
Mortality from 0 LTC
Mortality from 1 LTC
Mortality from 2 LTCs
Mortality from 3 LTCs
Mortality from 4 LTCs
Mortality from 5 LTCs
Mortality from 6 LTCs
Mortality from 7 LTCs
Mortality Rate (per person-year, log scale)
Age (years)
White Males (tfl capped at 15 years)
Greater than 100,000
52,000 to 100,000
31,000 to 51,999
18,000 to 30,999
Less than

Figure S7. Predicted transition rates of LTC accumulation and morality by family income

Predicted rates are shown on log scale for transitions between LTC states (upper panel) and mortality from each LTC state (lower panel) across ages 60-75 years. Income categories are represented by different colored lines. Predictions were generated for white males at calendar year 2004.

Abbreviations: LTCs, long-term conditions

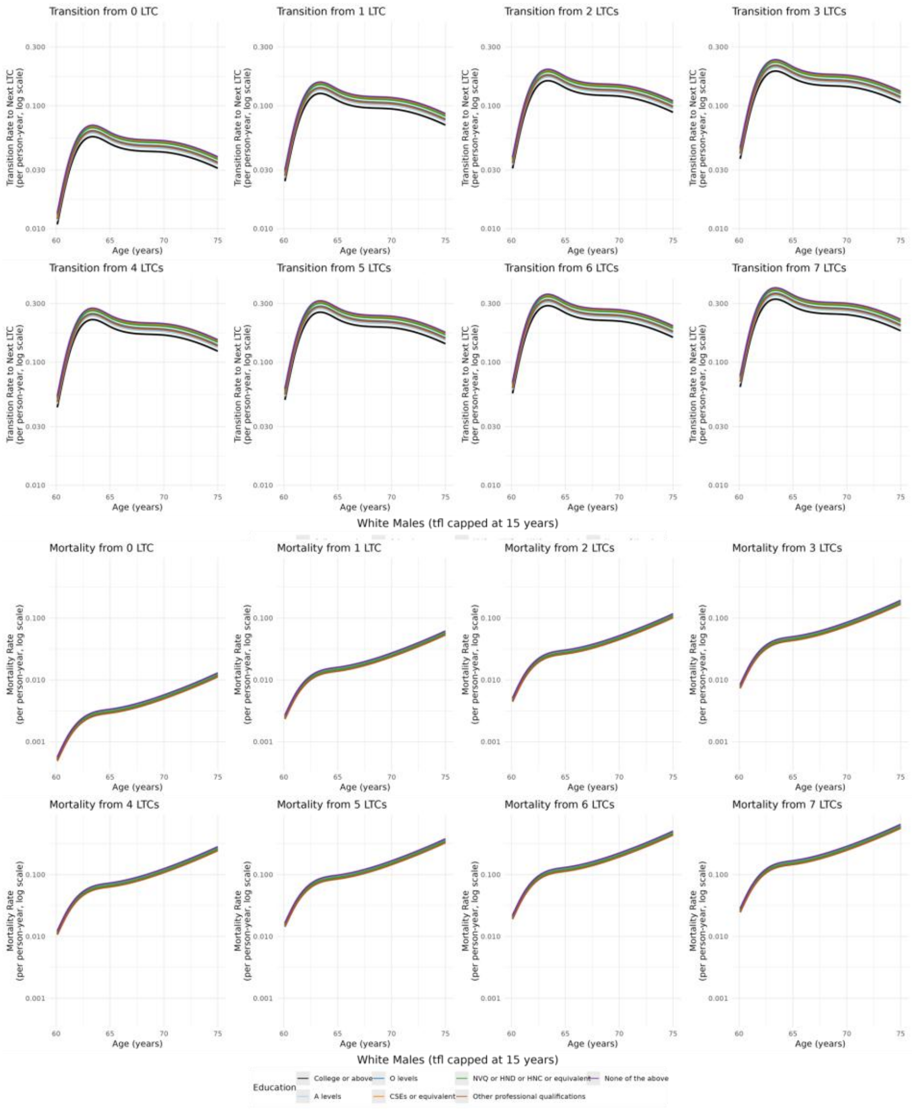

Figure S8. Predicted transition rates of LTC accumulation and mortality by education

Predicted rates are shown on log scale for transitions between LTC states (upper panel) and mortality from each LTC state (lower panel) across ages 60-75 years. Education categories are represented by different colored lines. Predictions were generated for white males at calendar year 2004.

Abbreviations: LTCs, long-term conditions

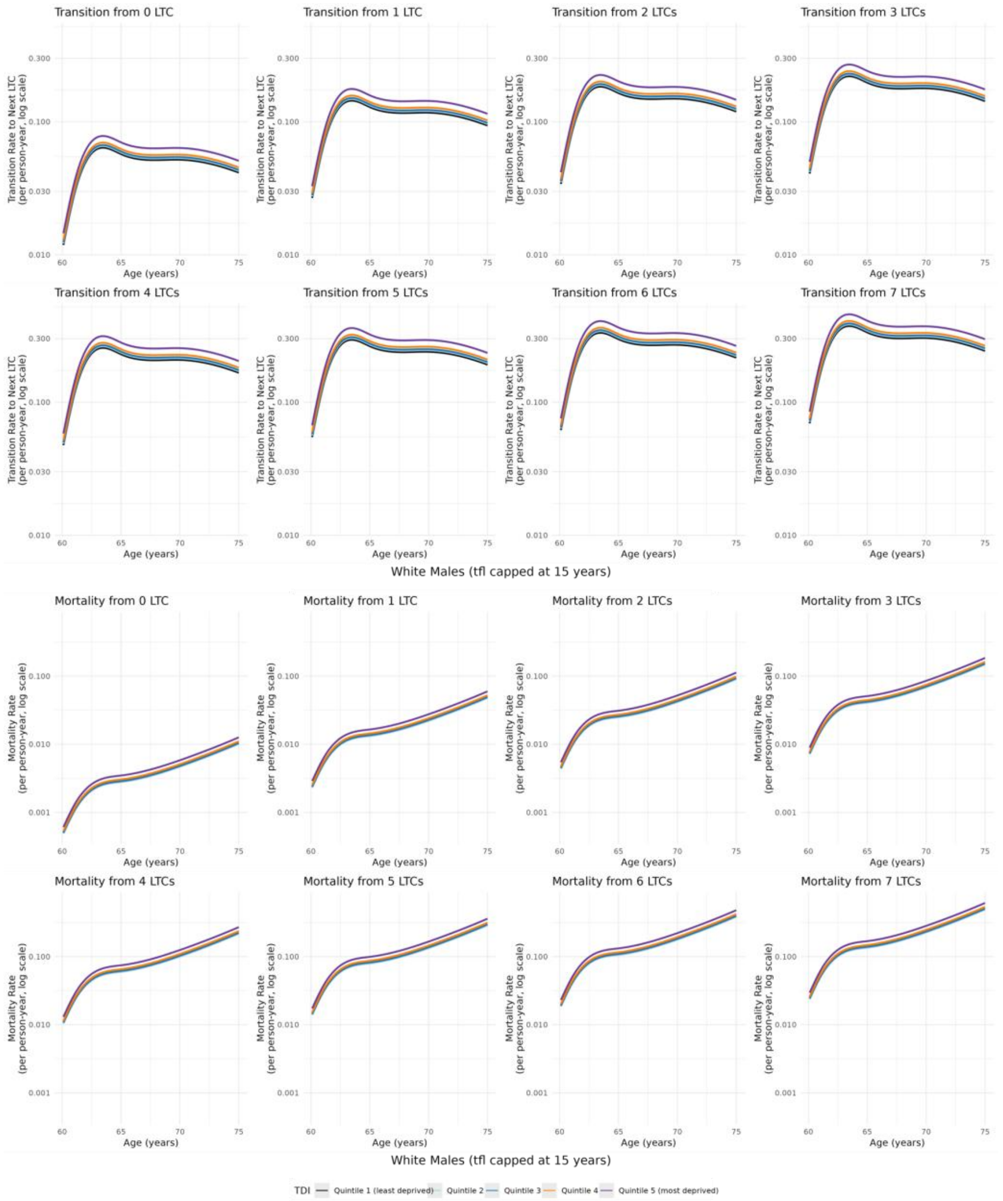
Transition from 0 LTC
Transition from 1 LTC
Transition from 2 LTCs
Transition from 3 LTCs
Transition from 4 LTCs
Transition from 5 LTCs
Transition from 6 LTCs
Transition from 7 LTCs
Transition Rate to Next LTC (per person-year, log scale)
Age (years)
White Males (tfl capped at 15 years)
Mortality from 0 LTC
Mortality from 1 LTC
Mortality from 2 LTCs
Mortality from 3 LTCs
Mortality from 4 LTCs
Mortality from 5 LTCs
Mortality from 6 LTCs
Mortality from 7 LTCs
Mortality Rate (per person-year, log scale)
Age (years)
White Males (tfl capped at 15 years)
TDI
Quintile 1 (least deprived)
Quintile 2
Quintile 3
Quintile 4
Quintile 5 (most deprived)

Figure S9. Predicted transition rates of LTC accumulation and morality by TDI

Predicted rates are shown on log scale for transitions between LTC states (upper panel) and mortality from each LTC state (lower panel) across ages 60-75 years. TDI levels are represented by different colored lines. Predictions were generated for white males at calendar year 2004.

Abbreviations: LTCs, long-term conditions; TDI, townsend deprivation index

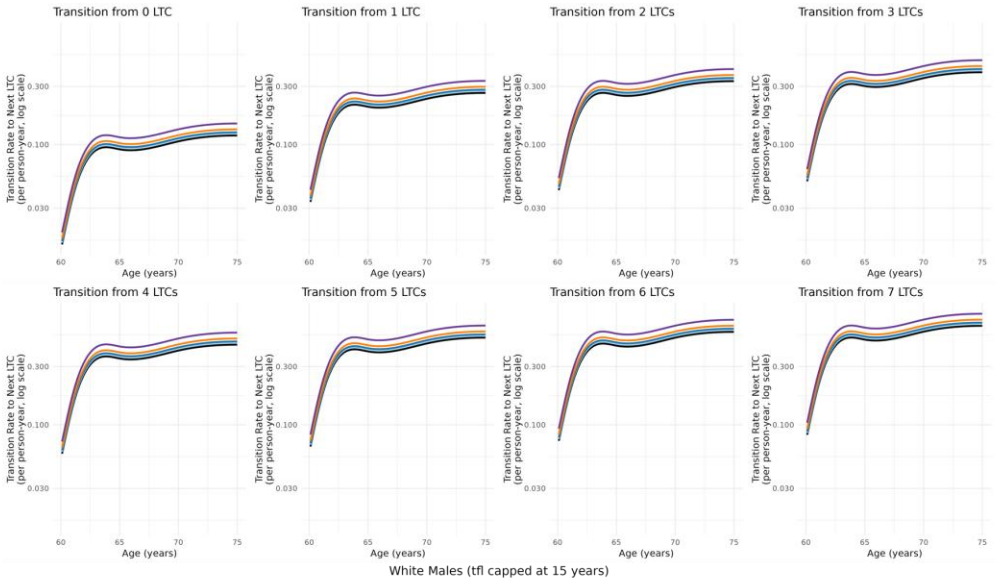
Transition from 0 LTC
Transition from 1 LTC
Transition from 2 LTCs
Transition from 3 LTCs
Transition from 4 LTCs
Transition from 5 LTCs
Transition from 6 LTCs
Transition from 7 LTCs
Transition Rate to Next LTC (per person-year, log scale)
Age (years)
White Males (tfl capped at 15 years)

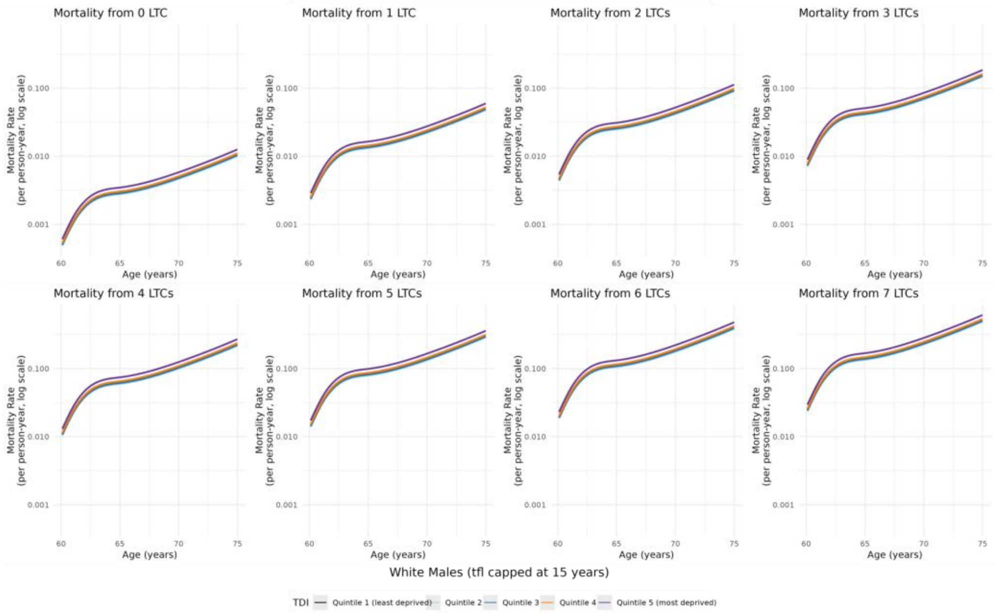
Mortality from 0 LTC
Mortality from 1 LTC
Mortality from 2 LTCs
Mortality from 3 LTCs
Mortality from 4 LTCs
Mortality from 5 LTCs
Mortality from 6 LTCs
Mortality from 7 LTCs
Mortality Rate (per person-year, log scale)
Age (years)
White Males (tfl capped at 15 years)
TDI
Quintile 1 (least deprived)
Quintile 2
Quintile 3
Quintile 4
Quintile 5 (most deprived)

Figure S10. Predicted transition rates of LTC accumulation and morality by IMD

Predicted rates are shown on log scale for transitions between LTC states (upper panel) and mortality from each LTC state (lower panel) across ages 60-75 years. IMD levels are represented by different colored lines. Predictions were generated for white males at calendar year 2004.
Abbreviations: LTCs, long-term conditions; IMD, index of multiple deprivation
