# Supplementary material for: Relationship between socioeconomic inequality and multimorbidity progression in UK Biobank data
Source: Commun Med (Lond). 2026 May 5;6:387. doi: 10.1038/s43856-026-01607-5 (PMC13346834; doi:10.1038/s43856-026-01607-5)
Supplement: Supplementary file 2 — Description of Additional Supplementary files [file 43856_2026_1607_MOESM2_ESM.docx]

**Description of Additional Supplementary Files**

Supplementary Data 1. Definition for codes list of 80 Long-term Conditions in the UK Biobank

Supplementary Data 2. Distribution of same-day diagnosis clusters based on ICD-10 coded long-term conditions in Hospital Episode Statistics (HES)

Supplementary Data 3. Transition matrix of LTC states and mortality in the study population

Supplementary Data 4. Characteristics of participants by Long-term Chronic Conditions in the UK Biobank

Supplementary Data 5. Range of age, period and person-years by LTCs in the UK Biobank

Supplementary Data 6. Transition Rate Ratios of LTCs accumulation and Mortality in Multistate Models

Supplementary Data 7. Transition rate ratios for LTC accumulation and mortality by family income

Supplementary Data 8. Transition rate ratios for LTC accumulation and mortality by education

Supplementary Data 9. Transition rate ratios for LTC accumulation and mortality by Townsend Deprivation Index

Supplementary Data 10. Transition rate ratios for LTC accumulation and mortality by Index of Multiple Deprivation

Supplementary Data 11. Transition-specific rate ratios for LTCs accumulation and mortality by SES indicators

Supplementary Data 12. Model-predicted morbidity state transition ages corresponding to a 20% probability by SES groups

Supplementary Data 13. Transition Rate Ratios for Family Income with LTC accumulation Stratified by Sex

Supplementary Data 14. Transition Rate Ratios for Education with LTC accumulation Stratified by Sex

Supplementary Data 15. Transition Rate Ratios for Townsend Deprivation Index with LTC accumulation Stratified by Sex

Supplementary Data 16. Transition Rate Ratios for Index of Multiple Deprivation with LTC accumulation Stratified by Sex

Supplementary Data 17. Frequency and proportion of multi-condition state transitions (jumps) in disease accumulation

Supplementary Data 18. Top 15 most common disease combinations diagnosed on the same calendar date
